# Gut microbiome and metabolome analyses reveal the protective effect of special high‐docosahexaenoic acid tuna oil on d‐galactose‐induced aging in mice

**DOI:** 10.1002/fsn3.2978

**Published:** 2022-07-15

**Authors:** Jing Zhang, Congmin Yi, Jiaojiao Han, Tinghong Ming, Jun Zhou, Chenyang Lu, Ye Li, Xiurong Su

**Affiliations:** ^1^ State Key Laboratory for Quality and Safety of Argo‐products Ningbo University Ningbo China; ^2^ School of Marine Science Ningbo University Ningbo China; ^3^ Faculty of Food Science Zhejiang Pharmaceutical College Ningbo China

**Keywords:** aging, gut microbiome, metabolome, protective effect, special high‐DHA tuna oil

## Abstract

Aging is closely related to altered gut function and its microbiome composition. To elucidate the mechanisms involved in the preventive effect of special high‐docosahexaenoic acid tuna oil (HDTO) on senescence, the effects of different doses of HDTO on the gut microbiome and metabolome of d‐galactose‐induced aging mice were studied. Deferribacteres and Tenericutes and uridine might be used as indicator bacteria and characteristic metabolites to identify aging, respectively. HDTO markedly improved the impaired memory and antioxidant abilities induced by d‐galactose. At the phylum level, the abundance of Firmicutes and Tenericutes was significantly increased upon d‐galactose induction, while that of Bacteroidetes, Proteobacteria, and Deferribacteres was significantly decreased. At the genus level, the variation mainly presented as an increase in the abundance of the Firmicutes genera *Ligilactobacillus*, *Lactobacillus*, and *Erysipelothrix*, the decrease in the abundance of the Bacteroidetes genera *Bacteroides* and *Alistipes*, the Firmicutes genus *Dielma*, and the Deferribacteres genus *Mucispirillum*. HDTO supplementation reversed the alterations in the intestinal flora by promoting the proliferation of beneficial flora during the aging process; the metabolic pathways, such as glycine–serine–threonine metabolism, valine–leucine–isoleucine biosynthesis, and some metabolic pathways involved in uridine, were also partially restored. Furthermore, the correlation analysis illustrated an obvious correlation between gut microbiota, its metabolites, and aging‐related indices. Moreover, it is worth noting that the metabolic regulation by dietary intervention varied with different HDTO doses and did not present a simple additive effect; indeed, each dose showed a unique modulation mechanism.

## Graphical abstract

The effects of different doses of HDTO on the gut microbiome and metabolome of D‐galactose‐induced aging mice were studied. It is worth noting that the metabolic regulation by dietary intervention varied with different HDTO doses and did not present a simple additive effect; indeed, each dose showed a unique modulation mechanism.

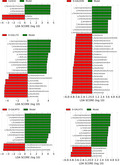

## 1 INTRODUCTION

Aging is an irreversible reality that cannot be relieved by most people. Aging is a natural process that cannot be prevented. The body's ability to adapt to the environment is progressively weakened, and its antioxidant capacity also decreases with age. The generation and removal of free radicals is out of balance, and health status is worsening, especially the decline in immune function (Campisi et al., 2019; Finkel & Holbrook, 2000). In addition, some studies have suggested that the composition and number of human gut microbiota are prominently altered during the aging process (Yamauchi et al., 2020). As a result, the risk of several diseases significantly increases, such as cognitive impairment, cardiovascular diseases, diabetes, and cancer (Ahima, 2009; Drew, 2018; Wang & Bennett, 2012). However, the current aging has indeed become the most important trend in the change of the world's population age structure; according to statistics, the elderly population tripled from 4% to 13% in the last century and is expected to grow sharply to reach 20% of the population by 2025 and 33% by 2050 (Azman & Zakaria, 2019; Sander et al., 2015), which will bring tremendous pressure and unprecedented challenges to the world economy. Thus, carrying out research on delaying aging and reducing age‐related diseases, continuously deepening the understanding of the underlying mechanisms of the aging process, and finding effective antiaging treatments and interventions are vital to the development of human health (Clark & Walker, 2018; Ragonnaud & Biragyn, 2021). Healthy aging constitutes a real economic challenge for nations in the 21st century.

Currently a reasonable diet and balanced nutrition are the most effective ways to suppress aging (Juárez‐Fernández et al., 2020). Studies have shown that adherence to a healthier diet may contribute to better physical, cognitive, and mental health during old age (Nijholt et al., 2016). Furthermore, increasing dietary intake of blue fish and long‐chain (LC) n‐3 PUFAs may help delay the accumulation of age‐related deficits (García‐Esquinas et al., 2019). Fish oil diet may be one of the best options for delaying aging and preventing various aging‐related diseases. Thus, the use of fish oil has increased in recent years.

Fish oil is a rich source of n‐3 polyunsaturated fatty acids (PUFAs), particularly docosahexaenoic acid (DHA) and eicosapentaenoic acid (EPA), which are dietary fats with an array of health benefits (Jamshidi et al., 2020; Su et al., 2008). Among them, DHA is a key component of all cell membranes, and is found in abundance in the brain and retina. It can improve the fluidity of neuronal cell membranes, thereby affecting signal transduction of neuronal cells. By preventing macular degeneration, Alzheimer's disease, Parkinson's disease, and other brain diseases, while enhancing memory and neuroprotection, DHA can play an important role in ensuring healthy aging (Cardoso et al., 2016). Furthermore, there is evidence that habitual consumption of fish may reduce the risk of cognitive decline, dementia, and Alzheimer's disease (Cunnane et al., 2009). Tuna oil is a typical DHA‐rich fish oil, and the tuna oil employed in this study is a further concentrated tuna oil with a higher DHA content. The potential mechanism of dietary intervention with high DHA tuna oil to suppress aging was preliminarily explored from the changes in the gut microbiome and metabolome of mice with mimetic aging induced by d‐galactose.

## 2 MATERIALS AND METHODS

### 2.1 Novel special high‐docosahexaenoic acid tuna oil source

High‐docosahexaenoic acid tuna oil (HDTO) utilized in the study was obtained from Ningbo Today Food Co., Ltd. Its fatty acid profile was determined by gas chromatography–mass spectrometry (GC‐MS; Agilent 7890/M7‐80EI system with a DB‐WAX column [60 m × 0.25 mm × 0.25 μm]) (Zhang et al., 2020).

### 2.2 Animals and treatment

Seventy‐two ICR mice (4‐ to 5‐week‐old males) with an average weight of 23.2 ± 2.1 g were purchased from the Laboratory Animal Center of Zhejiang Province (SCXK [Zhejiang] 2014‐0001). All experiments were performed in accordance with the Guide for the Care and Use of Laboratory Animals prepared by the Ningbo University Laboratory Animal Center (affiliated with the Zhejiang Laboratory Animal Common Service Platform) and experimental procedures and animal care were performed. All animal protocols were approved by the Ningbo University Laboratory Animal Center under permit number SYXK (ZHE 2008‐0110).

After 1 week of acclimatization, 72 male ICR mice were randomly assigned to six groups: control group (Control), aging model group (Model), positive drug group (d‐GALDON), low‐dose HDTO treatment group (d‐GALLTO), medium‐dose HDTO treatment group (d‐GALMTO), and high‐dose HDTO treatment group (d‐GALHTO). Each group was housed at 23 ± 1°C, under a 12:12‐h light/dark cycle and 60 ± 5% relative humidity. The modeling experiment lasted for 6 weeks. The control group was intraperitoneally injected with saline (1000 mg/kg/day), and the model and each diet intervention group were intraperitoneally injected with d‐galactose (800 mg/kg/day). During the modeling process, the mice could obtain food and water normally. Both the control group and the model group were given saline (800 mg/kg/day) by gavage; the positive drug group was given donepezil (1.2 mg/kg/day) by gavage; in the low‐, medium‐, and high‐dose HDTO groups, 65, 260, and 820 mg/kg/day HDTO were administered intragastrically, respectively. In the last week of the experiment (the sixth week), nine mice were randomly selected to conduct the Morris water maze experiment to assess the alterations in spatial memory and working memory of the mice. At the same time, the feces of each group were collected and stored at −80°C for subsequent experiments.

### 2.3 Morris water maze test

#### 2.3.1 Hidden platform search experiments

Four marked points were set at equal distances on the upper edge of the barrel as the water entry point for the mice. The projection points of the four water entry points on the water surface and the bottom of the bucket divided the water surface and the bucket equally into four quadrants (I, II, III, and IV), and a circular platform with a diameter of 9 cm was hidden in quadrant I, 2 cm below the water surface. In each test, the mouse head was placed toward the wall and randomly placed into the water from one of the other three quadrants outside the platform. The time required for the mouse to find the submerged platform and stand on it was recorded. If the mouse found the platform and stayed on it for 10 s, its escape latency was recorded; if not, the escape latency was recorded as 90 s. Each mouse was put into the pool from the four water entry points as one training session, and the interval between the two training sessions was 15–20 s, which lasted for 5 days.

#### 2.3.2 Spatial search memory experiments

On the sixth day, the platform was removed and the mice were placed in the water maze in the quadrant opposite to the previous location of the platform. The time and distance that the mice spent in the target quadrant and the number of times the mice traversed the former location of the platform were recorded.

### 2.4 Biochemistry test of the serum

Blood was collected from the orbital plexus and the serum was further isolated by centrifugation at 1500 *g* at 4°C for 15 min and then stored at −80°C. Serum levels of high‐density lipoprotein cholesterol (HDL‐C), low‐density lipoprotein cholesterol (LDL‐C), total cholesterol (TC), and malondialdehyde (MDA), as well as the activities of catalase (CAT) and superoxide dismutase (SOD) were measured using commercially available kits (Nanjing Jiancheng Bioengineering Institute) according to the manufacturer's instructions.

### 2.5 Gut microbiota composition and data analysis

Fecal samples were sent to LC‐Bio for 16S rRNA gene sequencing. The V_3_–V_4_ region of the 16S rRNA gene was amplified using primers 338F (5′‐ACTCCTACGGGAGGCAGCAG‐3′) and 806R (5′‐GGACTACHVG GGTWTCTAAT‐3′). The samples were sequenced using an Illumina MiSeq platform. Sequences with ≥97% similarity were assigned to the same operational taxonomic unit (OTU).

Alpha and beta diversity analyses were conducted using QIIME (version 1.8.0). Linear discriminant analysis (LDA) scores derived from the LDA effect size (LEfSe, https://huttenhower.sph.harvard.edu/galaxy/root?tool_id=lefse_upload) were used to identify the specific bacteria (*p* < .05, LDA score > 3.6) (Segata et al., 2011). Correlation analysis was conducted using Spearman's correlation in R software (version 3.6.3).

### 2.6 Metabolome analysis

#### 2.6.1 Extraction and derivatives of fecal metabolites

Fecal samples were dried with a freeze dryer (Labconco) and then smashed. Fifty milligrams of smashed stool was placed in 2‐ml microcentrifuge tubes, followed by the addition of 400 μl phosphate‐buffered saline (PBS; 1.9 mM Na_2_HPO_4_, 8.1 mM NaH_2_PO_4_, 150 mM NaCl, pH 7.4). After 2 min of vortex mixing, the mixture was homogenized for 10 min using an ultrasonic homogenizer. The fecal slurry was centrifuged for 20 min at 4°C (26,000×*g*), and 200 μl of the supernatant was transferred to a dry EP tube. Then, 400 μl of ice methanol was added to the precipitate, which was vortexed for 10 min. After centrifugation at 26,000×*g* for 10 min, 200 μl of supernatant was pipetted and combined with the first 200 μl of supernatant, mixed well, and centrifuged. Finally, 300 μl of the mixture was transferred into a glass vial, and 2 μl of tetracosane standard solution was added, and the solvent was evaporated under a stream of nitrogen, followed by trimethylsilyl derivatization before GC‐MS analysis (Pechlivanis et al., 2014).

#### 2.6.2 Fecal metabolic profiling

Analyses were conducted using an Agilent 6890/7000C Triple Quadrupole (Agilent) equipped with an HP‐5MS fused‐silica capillary column (30 m × 250 μm i.d.; Agilent J&W Scientific). The injection volume was 2 μl in split mode (split ratio 1:10), and the solvent delay time was set to 3.5 min. The initial oven temperature was maintained at 60°C for 3 min, increased to 200°C at a rate of 5°C/min, maintained for 1 min, then increased at 5°C/min to 300°C and maintained for 10 min. The temperatures of the injector, transfer line, and electron impact (EI) ion source were set to 280°C, 250°C, and 280°C, respectively. The electron energy was 70 eV, and mass data were collected in full scan mode (m/z 50–800).

Pretreatment of the resulting MS data. The metabolites were identified using the NIST database. After removing any known pseudo‐positive peaks from the datasets, such as peaks caused by noise, column bleed, and *N*‐methyl‐*N*‐(trimethylsilyl)‐trifluoroacetamide (MSTFA) derivatization procedure, a CSV file was obtained that listed m/z and retention time with corresponding intensities for each metabolite from every sample in the positive dataset. An internal standard (tetracosane‐*n*‐heptane) calibration was used to reduce the deviation between individual samples. Finally, the normalized dataset was imported into SIMCA‐P software (v14.1, Umetric) for multivariate statistical analysis and plotting of fecal metabolite profiles, where supervised partial least squares discrimination analysis (PLS‐DA/OPLS‐DA) was applied to identify metabolites that significantly contributed to the classification. Metabolic features with variable importance in projection (VIP) values of >1.0, and *p* < .05 in the OPLS‐DA model were considered to be significantly different metabolites in the paired comparison. The metabolites selected above were subjected to enrichment analysis and pathway topology analysis using MetaboAnalyst (http://www.metaboanalyst.ca) to determine biologically meaningful metabolic patterns and the most impacted pathways.

### 2.7 Statistical analysis

Data are shown as means ± standard error of the mean (SEM) unless otherwise noted, and the data were analyzed using SPSS 23.0, GraphPad Prism version 8.0, and OriginPro software. Student's *t* test was used to identify differences between the two groups. For data whose distribution did not conform to the Gaussian model of heterogeneity, nonparametric Kruskal–Wallis analysis was employed. Differences were considered statistically significant at *p* < .05.

## 3 RESULTS

Aging is a complex process that is accompanied by the occurrence of multiple chronic diseases. Although it is inevitable, researchers are still trying to understand the underlying mechanism of this process and determine possible ways to regulate aging to delay its consequences (Kolovou et al., 2014). DHA is a type of *n*‐3 polyunsaturated fatty acid that has drawn much attention and has a significant effect on counteracting aging and improving cognition. Decreased DHA levels are associated with cognitive decline during aging (Cardoso et al., 2016). Loss of DHA from nerve cell membranes can result in central nervous system dysfunction, anxiety, irritability, dyslexia, impairment of memory and cognitive functions, and prolonged reaction time (Gow & Hibbeln, 2014; Hellhammer et al., 2012; Ross, 2009; Stonehouse et al., 2013). However, DHA cannot be synthesized by the human body, and therefore must be obtained from the diet. Fish oil diet is a recommended way to replenish DHA in modern diets. Tuna oil is a typical DHA‐rich fish oil. Nevertheless, the causal relationship between fish oil supplementation and cognitive function improvement is still unclear and further research is needed (Daiello et al., 2015). Therefore, we explored the effects of HDTO diet intervention on the changes in intestinal flora and metabolome of d‐galactose‐induced aging mice by supplementation with HDTO.

### 3.1 Effects of HDTO on the behavior of aging mice

**FIGURE 1:**
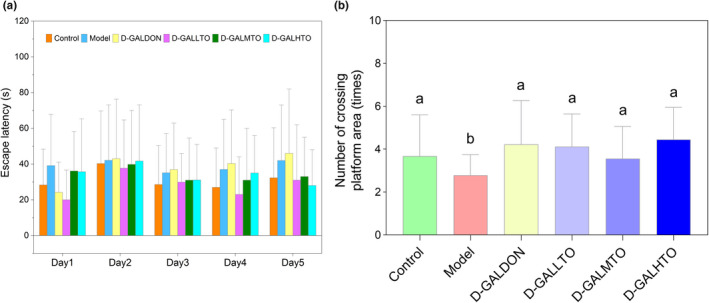
Spatial memory measures based on Morris water maze test. (a) Escape latency to the platform; (b) number of crossing in the target area (Different letters on the bar chart correspond to statistically significant differences (P<.05) between two groups). The values are expressed as the mean ± SEM

The Morris water maze test was conventionally employed to determine the spatial learning and memory abilities of mice. As indicated in Figure 1a, the 5‐day continuous testing revealed that the escape latency of d‐galactose‐induced aging mice to the platform was markedly longer than that of control mice (*p* < .05), while the platform area crossing times were obviously reduced after the platform was removed (*p* < .05). These results suggest that administration of d‐galactose significantly impaired the spatial memory and learning ability of mice. Compared with the model group, the escape latency of the mice with HDTO diet intervention was correspondingly shortened. Nonetheless, with the increase in fish oil supplement dose, the shortening of escape latency did not reflect a dose‐dependent relationship. Mice administered with a positive drug showed a reduction in escape latency only on the first day. Meanwhile, the results in Figure 1b indicate that when the aging mice were treated with HDTO and a positive drug, their platform area crossing times were obviously increased, and the time was increased from 3 to more than 4. However, there was no significant difference between the intervention and treatment effects, and the three doses of fish oil supplementation did not show a dose‐dependent reversal effect. The above results suggested that the aging model was well constructed, and HDTO diet intervention improved the spatial learning and memory ability of d‐galactose‐induced aging mice.

### 3.2 Effects of HDTO on serum lipids and oxidative stress in d‐galactose‐induced mice

Serum lipid and lipoprotein levels increase with age, and accumulating studies have shown that oxidative stress is closely related to biological aging (Liu et al., 2020; Zhao et al., 2018). Given that, the activities of SOD and CAT, as well as TC, HDL‐C, LDL‐C, and MDA levels in d‐galactose‐induced mice were determined. Compared with the control group, d‐galactose injection led to a significant increase in the levels of LDL‐C and TC in the serum of mice (*p* < .05), and a decrease in the level of HDL‐C. Then, in comparison to the aging group, HDTO dietary supplementation significantly reduced the LDL‐C level (*p* < .05), and the TC level also decreased, but only when the HDTO dose reached the highest level had a remarkable effect (*p* < .05) and was restored to a level similar to that of the control group. Positive drug treatment also caused a significant increase and decrease in HDL‐C and LDL‐C levels (*p* < .05), but the TC level increased insignificantly.

**FIGURE 2:**
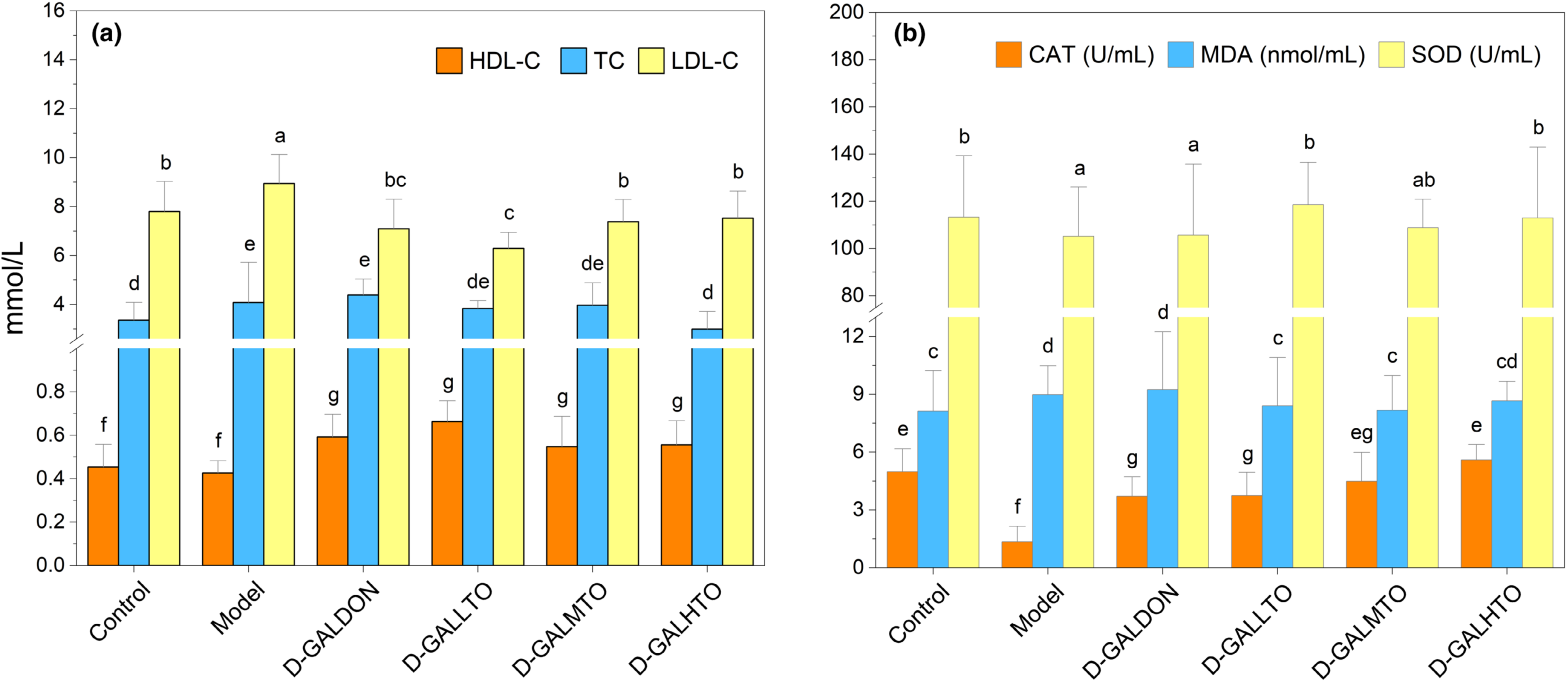
Effects of special high‐DHA tuna oil on serum lipid metabolism and oxidation activity in the d‐galactose‐induced mice (a: lipid level; b: antioxidant activity). The different letters marked on every group represent significant differences between different groups (*p* < .05). The values are expressed as the mean ± SEM

The results of the oxidation biomarkers in Figure 2b showed that in comparison with the control group, the MDA content of the model group mice was significantly elevated, and the activity of CAT and SOD were significantly reduced, and the activity of CAT was reduced by nearly three times. HDTO supplementation reversed the levels of these serum indicators in d‐galactose‐induced aging mice. All three doses of HDTO restored SOD activity to the level of the control group, but medium‐dose HDTO had no significant effect. With the increase in HDTO dose, CAT activity gradually restored to the level of the control group (*p* < .05), and the MDA content was reduced by 6.67%, 8.89%, and 3.33%, respectively, under the three HDTO doses, which did not comply with the dose effect. Positive drug treatment had a significant reversal effect on CAT activity (*p* < .05), and SOD activity increased marginally, but the increase in MDA content induced by d‐galactose could not be suppressed. These results indicate that HDTO exhibits significant hypolipidemic and antioxidant effects in d‐galactose‐induced aging mice, but these effects did not conform to the dose effect.

### 3.3 Effects of HDTO on gut microbiota in d‐galactose‐induced mice

**FIGURE 3:**
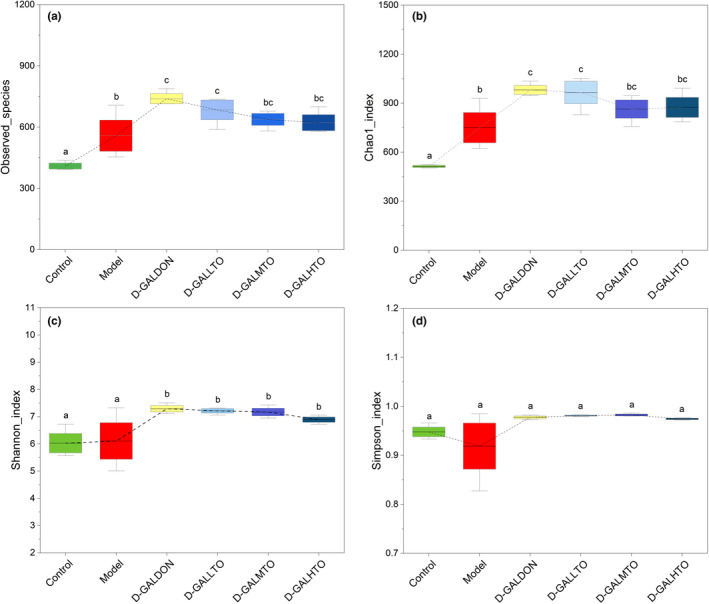
Structure analysis of intestinal flora in the mice. Alpha diversity analysis of (a) observed species, (b) Chao 1, (c) Simpson, and (d) Shannon indices. The different letters represent significant differences between different groups (*p* < .05).The values are expressed as the mean ± SEM

d‐galactose injection led to a natural aging status but did not cause a decline in the abundance of the intestinal flora of mice. The observed species and Chao1 index increased significantly (Figure 3a,b). Donepezil administration further increased the observed species and Chao1 index. In the three HDTO diet intervention groups, with the increase in HDTO dose, the observed species and Chao1 index showed a gradual decreasing trend. In comparison to the control group, the Shannon index of the intestinal bacteria in the model group at the OTU level increased slightly, while the Simpson index decreased; however, compared with the model group, the positive drug treatment and the three HDTO diet intervention groups all further increased the Shannon and Simpson indices; however, with the increase in HDTO dose, the increasing extent of Shannon index gradually decreased, but statistical significance was not observed (Figure 3c,d).

**FIGURE 4:**
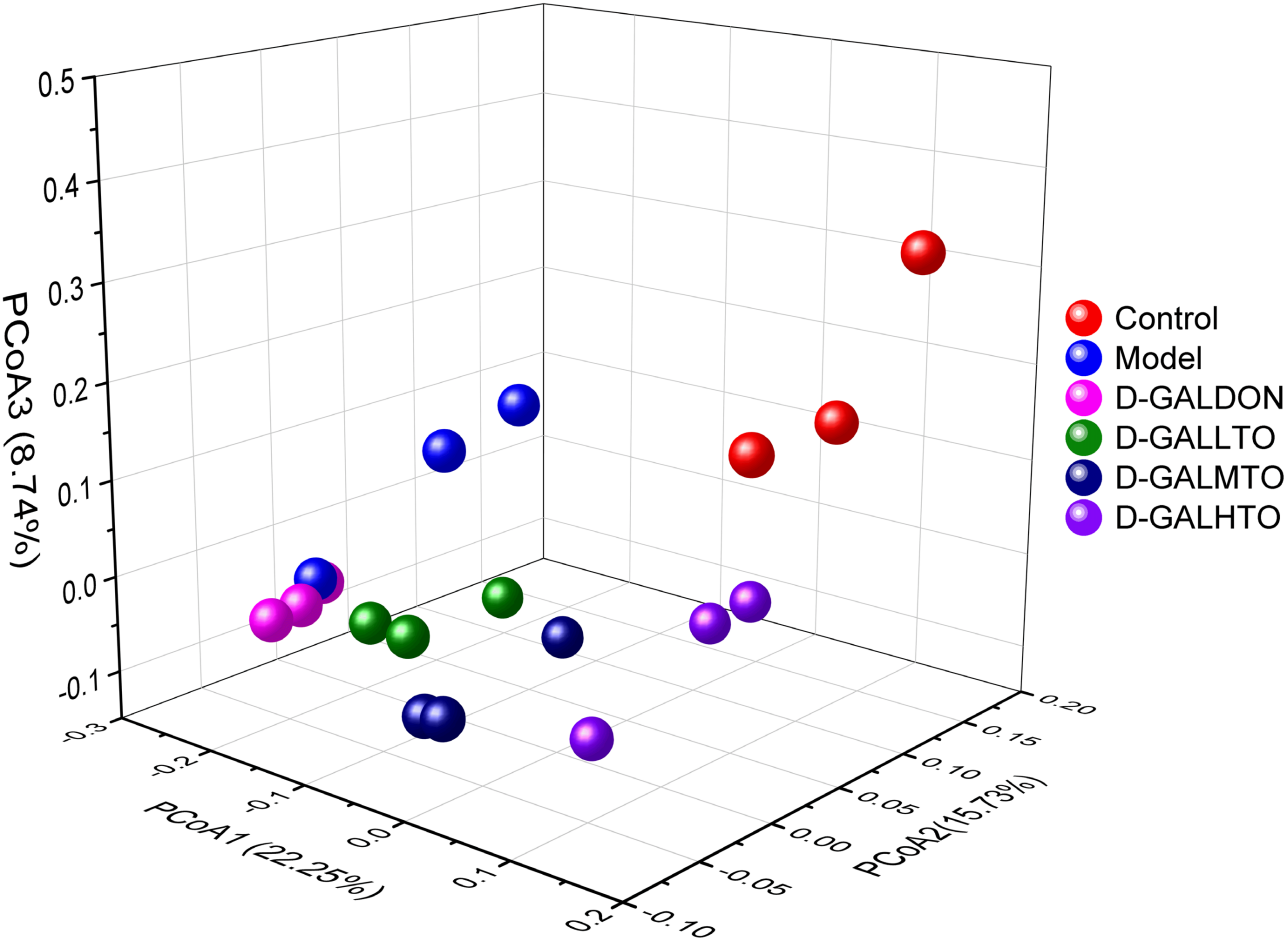
Principal coordinate analysis (PCoA) on the OTU level based on the unweighted Unifrac distance

As shown in Figure 4, the flora distribution in the control group, the model group, the positive drug treatment, and the three diet intervention groups were remarkably separated, suggesting that the bacterial composition of each group was different. Furthermore, the flora distribution of d‐galactose‐induced aging mice with low, medium, and high doses of HDTO diet intervention gradually approached that of the control group with the increase in the intervention dose. Therefore, the species composition and structure of the gut microbiota in the aging mice supplemented with high‐dose HDTO were more similar to those of the control group. From the perspective of spatial distribution, the flora distribution of the donepezil treatment group was closer to that of the model group. It can be seen from the above results that d‐galactose injection caused an increase in the abundance and diversity of gut microbiota in mice, but the 6‐week HDTO diet intervention failed to significantly restore these changes to that of the control. However, the structural composition of the intestinal bacteria of the mice in each group was significantly different, and the structural composition of the intestinal bacteria of the mice in the HDTO diet intervention groups was more similar to that of the control group.

**FIGURE 5:**
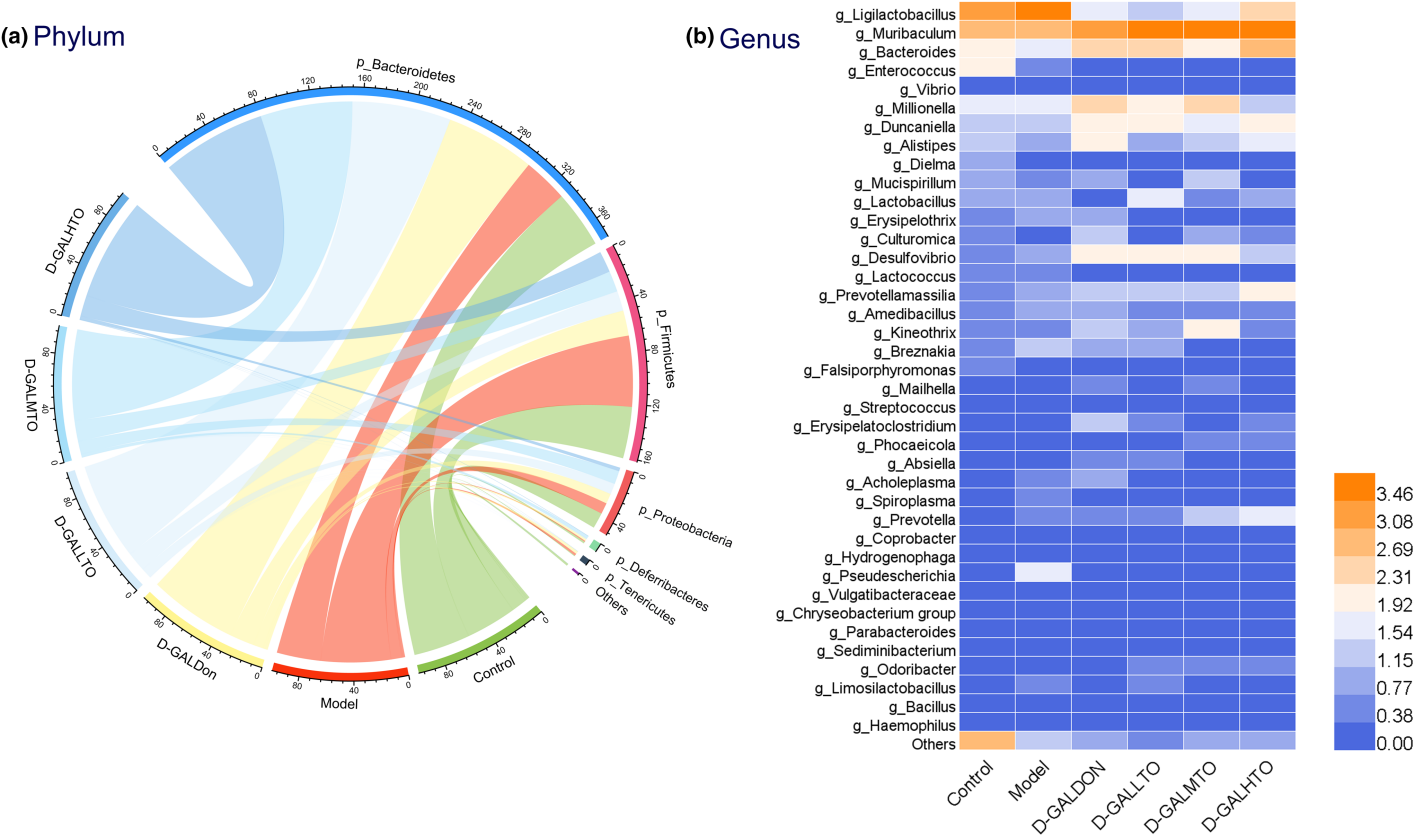
High‐docosahexaenoic acid tuna oil (HDTO) supplementation alters gut microbiota structure in the aging mice. Relative abundance of the bacterial profile at phylum level (a) and at the genus level (b). The values are expressed as the mean ± SEM

The alteration in the intestinal flora of each group due to differences in diet was further analyzed at the species level (Figure 5). At phylum level (Figure 5a), the dominant bacterial community in the control, model, positive‐drug treatment, and three HDTO dietary intervention groups included Bacteroidetes (the relative abundances were 44.33%, 34.55%, 69.14% and 76.15%, 70.98%, 80.46%, respectively), Firmicutes (the relative abundances were 39.73%, 54.52%, 19.46% and 15.36%, 16.07%, 16.40%, respectively), Proteobacteria (the relative abundances were 12.23%, 8.56%, 7.80% and 8.30%, 10.22%, 2.77%, respectively), Deferribacteres (the relative abundances were 1.41%, 0.77%, 1.52% and 0.15%, 2.55%, 0.28%, respectively), and Tenericutes (the relative abundances were 0.62%, 1.60%, 2.08% and 0.03%, 0.17%, 0.06%, respectively). d‐galactose injection significantly decreased the abundances of Bacteroidetes, Proteobacteria, and Deferribacteres, while that of Firmicutes and Tenericutes significantly increased. Compared with the model group, HDTO diet intervention remarkably reversed the changes in Bacteroidetes, Firmicutes, and Tenericutes in the mice flora induced by d‐galactose, and even the extent of increase and decrease was more obvious, while the changes in the abundance of Proteobacteria and Deferribacteres reversed only at medium doses of HDTO. The positive drug treatment notably reversed the abundance changes of Bacteroidetes, Firmicutes, Deferribacteres, and Tenericutes.

**FIGURE 6:**
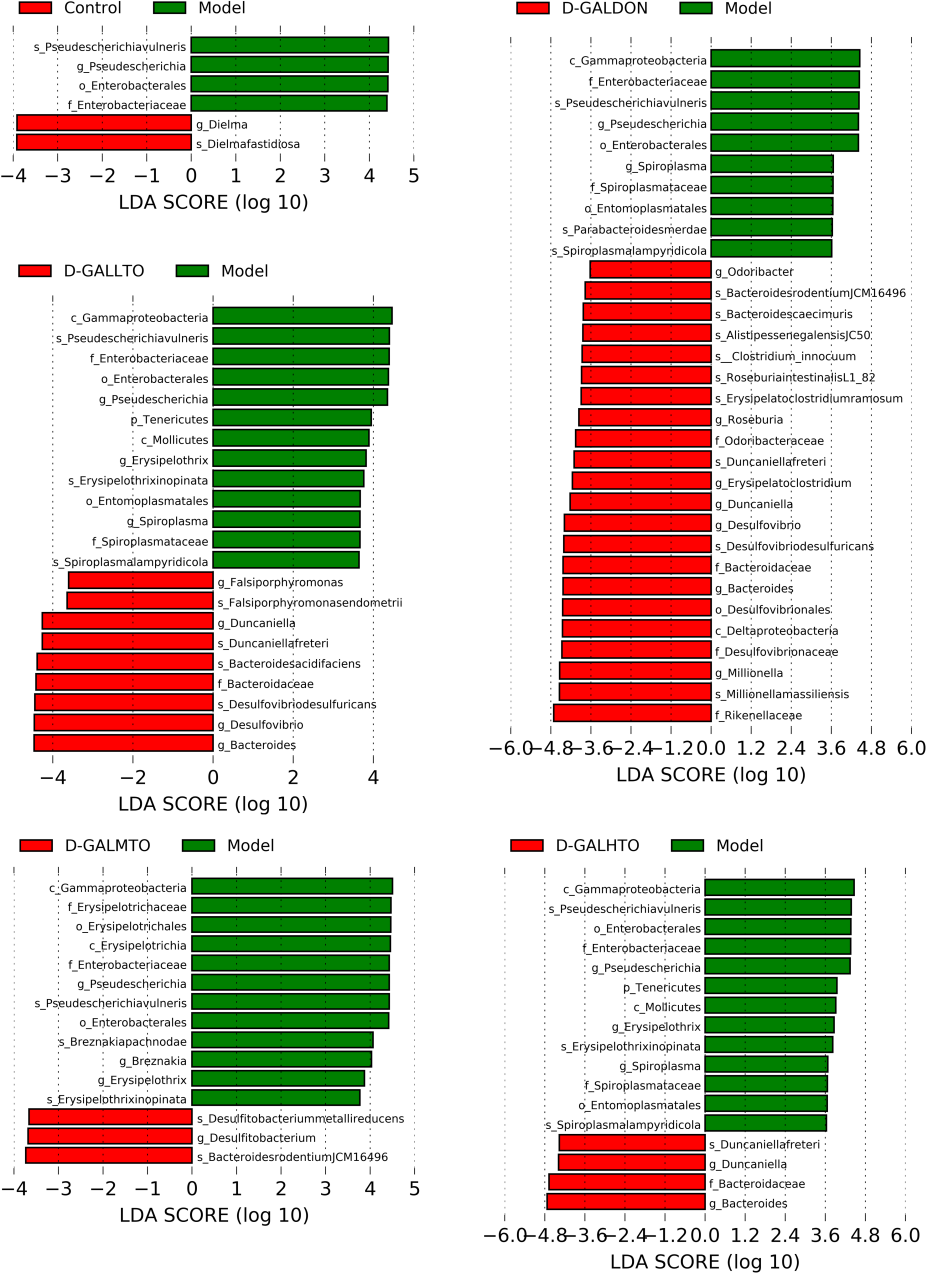
Linear discriminant analysis (LDA) coupled with effect size measurements identified the most differentially expressed taxa between two groups. Only taxa meeting an LDA significant threshold of >3.6 and *p* < .05 were shown

At the genus level, d‐galactose injection prominently elevated the abundance of *Ligilactobacillus*, *Lactobacillus*, and *Erysipelothrix*, and decreased that of *Bacteroides*, *Alistipes*, *Dielma*, and *Mucispirillum* in the gut microbiota of mice. Among them, only the abundance alterations in *Ligilactobacillus*, *Bacteroides*, *Alistipes*, and *Erysipelothrix* were reversed by HDTO diet intervention (Figure 5b). The effects of the positive drugs were similar. LDA effect size (LEfSe) analysis was further employed to identify specific phenotypes (with LDA scores >3.6) that significantly differed in response to d‐galactose or HDTO (Figure 6). Compared with the control group, the abundance of *Pseudescherichia* in the model group mice increased significantly, and *Dielma* decreased significantly; compared with the three HDTO diet intervention groups, the biomarkers with significant difference were *Pseudescherichia*, *Erysipelothrix*, *Spiroplasma*, *Falsiporphyromonas*, *Duncaniella*, *Desulfovibrio*, *Bacteroides*, *Breznakia*, and *Desulfitobacterium*. The positive drug administration caused a prominent alteration in the abundance of *Pseudescherichia*, *Spiroplasma*, *Odoribacter*, *Roseburia*, *Erysipelatoclostridium*, *Duncaniella*, *Desulfovibrio*, *Bacteroides*, and *Millionella* in the gut microbiota of d‐GALDON mice. These results indicated that HDTO supplementation significantly ameliorated the structure and composition of the gut microbiota in d‐galactose‐induced mice.

### 3.4 Effects of HDTO on fecal metabolite profiles in d‐galactose‐induced mice

**FIGURE 7:**
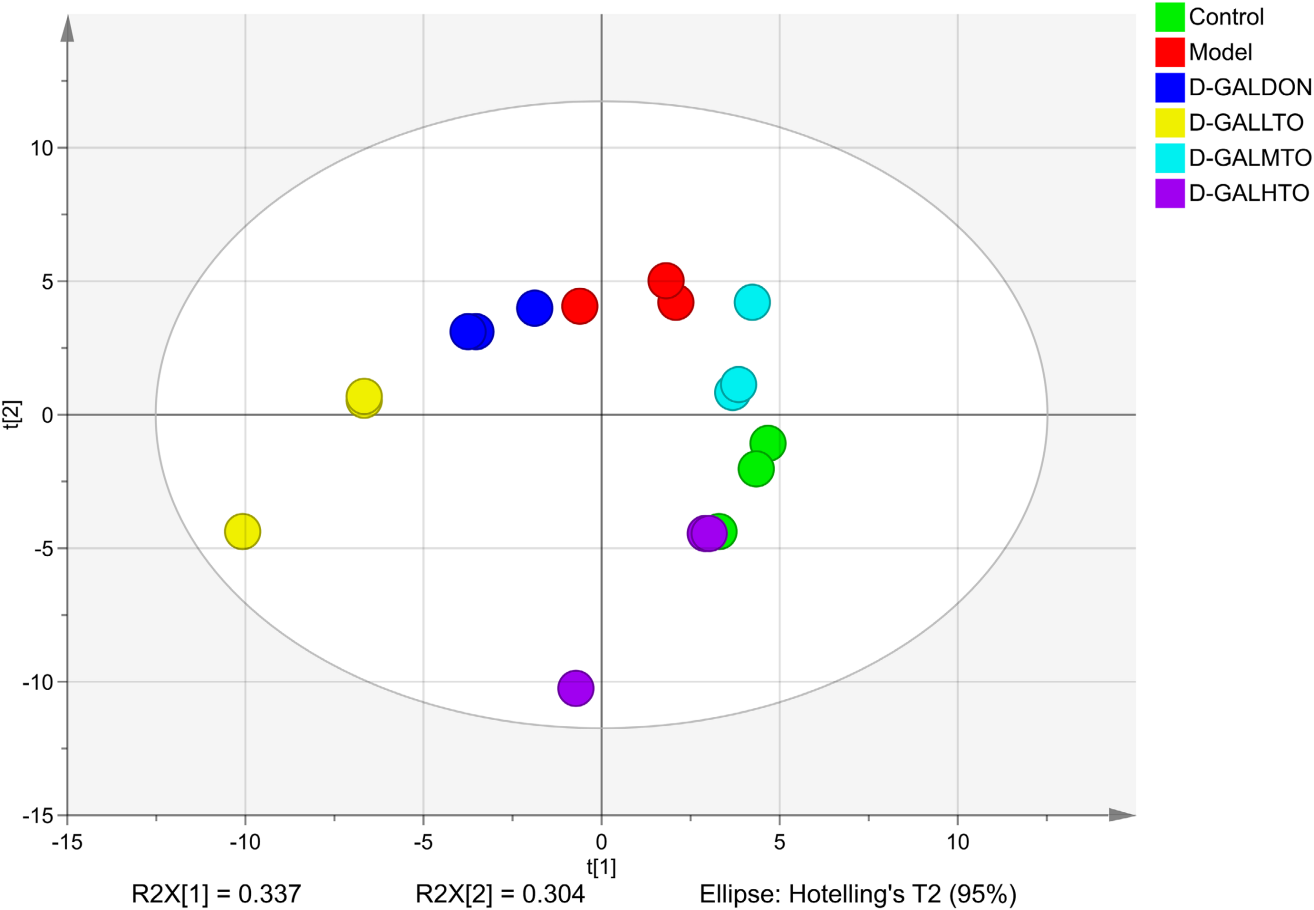
Partial least‐squares discriminant analysis (PLS‐DA) score plot based on GC‐MS/MS spectra of fecal sample among the groups of control, model, d‐GALDON, d‐GALLTO, d‐GALMTO, and d‐GALHTO

GC‐MS/MS was used to determine the metabolites in fecal samples. To confirm the effect of HDTO diet intervention on the metabolic pattern of d‐galactose‐induced aging mice, and to identify the metabolites that significantly changed, partial least squares discriminant analysis (PLS‐DA) and orthogonal partial least squares discriminant analysis (OPLS‐DA) was used to analyze the data measured by GC‐MS/MS. As shown in Figure 7, all the groups achieved separation from each other within the 95% confidence interval, and on the t2 axis, it could be discerned that HDTO diet intervention restored the metabolic disorders of aging mice. The metabolic characteristics of the d‐GALDON group were similar to those of the model group. OPLS‐DA analysis was further performed to ascertain the metabolites that had the greatest effect on clustering, and those metabolites with variable importance in the projection (VIP) value ≥1.5, and Student's *t* test *p* value <.05 were screened as the discriminating metabolites (Figure S1). Control, d‐GALDON, d‐GALLTO, d‐GALMTO, and d‐GALHTO were compared with the model group, and 49 metabolites were identified. They are mainly carbohydrates and amino acids. In comparison with the model group, HDTO diet intervention at dose of 65 mg/kg/day significantly elevated the concentration of d‐arabinose, d‐erythopentose, l‐5‐oxoproline, l‐serine, tyrosine, l‐valine, l‐threonine, l‐methionine, l‐leucine, inositol, glycerol, uridine, and uracil, and markedly decreased the content of l‐fucose, cellobiose, and glycine; HDTO diet intervention at dose of 260 mg/kg/day significantly elevated the concentration of d‐galactose, d‐xylose, d‐glucose, 2‐α‐mannobiose, l‐serine, l‐proline, l‐valine, l‐threonine, glycerol, uridine, and pyroglutamine acid, and markedly decreased the content of d‐arabinose, l‐fucose, cellobiose, l‐5‐oxoproline, l‐aspartic acid, glycine, and amphetamine; at intervention dose of 820 mg/kg/day, the significantly increased metabolites were d‐galactose, d‐xylose, d‐arabinose, d‐glucose, d‐rhamnose, l‐threonine, l‐methionine, uridine, uracil, pyrimidine, lactic acid, and succinic acid, while l‐5‐oxoproline, l‐aspartic acid, and glycine were those metabolites that were remarkably decreased.

Metabolic pathway analysis (MetPA) was performed using metaboanalyst 5.0 (*p* < .05). The occurrence of disorders of eight metabolic pathways might be associated with d‐galactose supplementation (Figure S2), namely aminoacyl–tRNA biosynthesis, valine–leucine–isoleucine biosynthesis, glycine–serine–threonine metabolism, pantothenic acid and CoA biosynthesis, glycolysis/gluconeogenesis, glutathione metabolism, alanine–aspartic acid–glutamic acid nucleotide metabolism, and acetaldehyde acid–dicarboxylic acid sugar metabolism. The metabolic pathways changed by positive drug treatment included aminoacyl–tRNA biosynthesis, valine–leucine–isoleucine biosynthesis, valine–leucine–isoleucine degradation, glycine–serine–threonine metabolism, pantothenic acid and CoA biosynthesis, galactose metabolism, glutathione metabolism, glyoxylic acid–dicarboxylate metabolism, and cysteine–methionine metabolism.

The effects of three doses of HDTO diet on the metabolic alterations in d‐galactose‐induced aging mice were compared. The metabolic pathways changed by all three doses were: aminoacyl–tRNA biosynthesis, pantothenic acid–CoA biosynthesis, glutathione metabolism, and glycine–serine–threonine metabolism. The metabolic pathways changed by low‐ and medium‐dose HDTO diet interventions included phenylalanine–tyrosine–tryptophan biosynthesis, glyoxylic acid–dicarboxylic acid esters metabolism; the metabolic pathways that are altered by medium‐ and high‐dose HDTO diet interventions were β‐alanine metabolism, alanine–aspartic acid–glutamate metabolism, and valine–leucine–isoleucine biosynthesis was a metabolic pathway that was altered by both low‐ and high‐dose HDTO. The metabolic pathways individually regulated by low‐dose HDTO diet intervention were galactose metabolism, cysteine–methionine metabolism, and valine–leucine–isoleucine degradation, and the pathways of amino sugar–nucleotide sugar metabolism and glycolysis/gluconeogenesis were regulated by medium‐ and high‐dose HDTO diet interventions, respectively. It can be seen from the above results that the metabolic regulation by HDTO diet intervention would vary with different doses, but by no means a simple additive effect. Each dose had its own regulatory mode. Moreover, the changes in metabolic pathways caused by positive drug treatment were all observed in d‐galactose‐induced aging mice with HDTO diet intervention.

### 3.5 Correlation analysis

#### 3.5.1 Correlation between gut biomarkers and lipid metabolism, oxidative stress parameters

**FIGURE 8:**
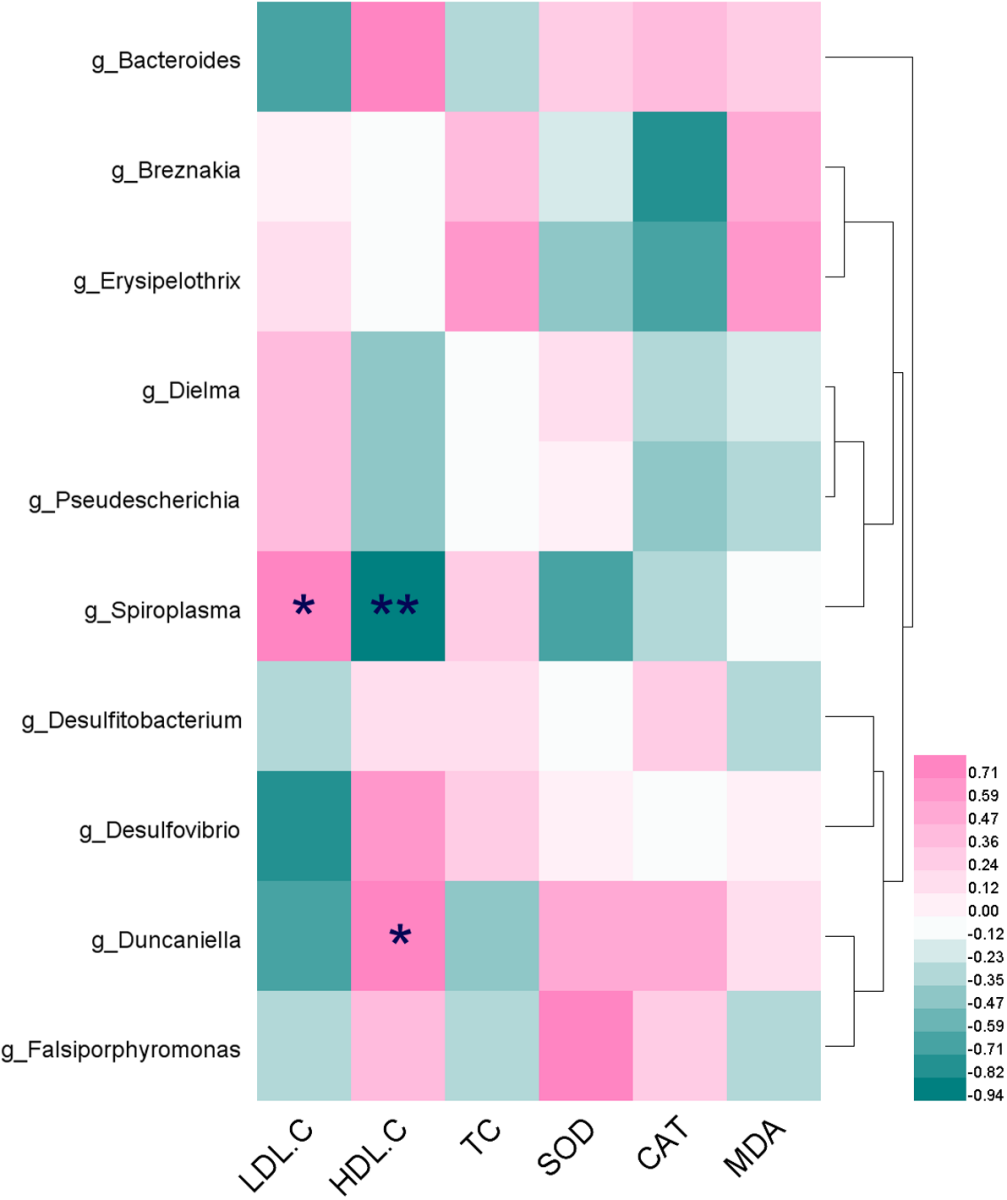
Spearman's correlations between lipid metabolism, oxidative stress parameters, and the abundance of bacterial species. *p < .05, **p < .01

The relationships between six biochemical indices and significantly different genera of gut microbiota were analyzed by establishing a correlation matrix to calculate Spearman's correlation coefficient. As indicated in Figure 8, for the serum lipid metabolism indices, LDL‐C was negatively correlated with *Bacteroides*, *Desulfovibrio*, and *Duncaniella* (|*r*| ≥ .6), while positively correlated with *Spiroplasma* (*r* = .82, *p* < .05); HDL‐C was positively correlated with *Bacteroides*, *Desulfovibrio*, and *Duncaniella* (*r* ≥ .6), and negatively correlated with *Spiroplasma* (*r* > .9, *p* < .01); TC was positively related to *Erysipelothrix* (*r* = .6). For the oxidative stress parameters, SOD was positively and negatively correlated with *Falsiporphyromonas* and *Spiroplasma* (|*r*| ≥ .6); CAT was negatively correlated with *Breznakia* and *Erysipelothrix* (|*r*| ≥ .6), while MDA was positively related to *Erysipelothrix* (*r* = .6).

#### 3.5.2 Correlation between gut biomarkers and significantly different metabolites

**FIGURE 9:**
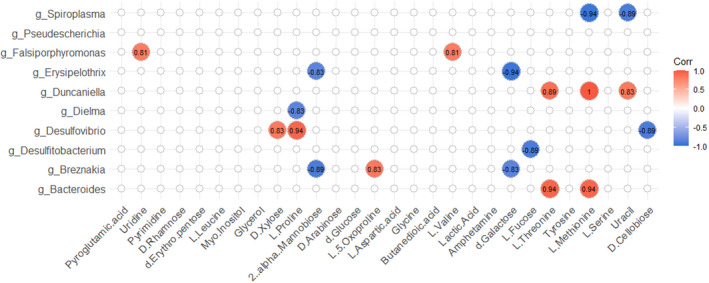
Spearman's correlations between the significantly differential metabolites and the abundance of bacterial species on the genus level (*p* < .05)

The correlation between 10 biomarkers of gut microbiota screened from the comparison between the control group, three doses of HDTO group, and the model group and 28 differential metabolites was analyzed. As shown in Figure 9, *Spiroplasma* was negatively correlated with l‐methionine and uracil; *Falsiporphyromonas* was positively correlated with l‐valine and uridine; *Erysipelothrix* was negatively correlated with 2‐α‐mannobiose and d‐galactose; *Duncaniella* was positively correlated with l‐threonine, l‐methionine, and uracil; *Dielma* and *Desulfitobacterium* were negatively correlated with l‐proline and l‐fucose, respectively; *Desulfovibrio* was positively correlated with d‐xylose and l‐proline, and negatively correlated with d‐cellobiose; *Breznakia* was negatively correlated with 2‐α‐mannose and d‐galactose, and positively correlated with l‐5‐oxoproline; *Bacteroides* was positively correlated with l‐methionine and l‐fucose.

## 4 DISCUSSION

The results of this study suggest that HDTO has a protective effect on lipid metabolism disorders, oxidative stress, and cognitive impairment in d‐galactose‐induced mimetic aging mice. In addition, the gut microbiota plays a vital role in the body and regulates health conditions and disease progression. As reported in the literature, the composition, diversity, and functional features of the intestinal microbiota of aging‐associated gut microbiota are altered (Vaiserman et al., 2017). Therefore, establishing nutritional strategies aimed at counterbalancing the specific alterations occurring in the microbiota has been proposed as a promising treatment for age‐related metabolic and neurodegenerative diseases (Bana & Cabreiro, 2019). Dietary interventions targeting the flora can regulate host health and aging by enhancing antioxidant activity, improving immune homeostasis, inhibiting chronic inflammation, regulating fat deposition and metabolism, and preventing insulin resistance (Clements et al., 2018; Cӑtoi et al., 2020). It was found in our study that d‐galactose caused an increase in the abundance of mouse gut microbiota, while its diversity was not markedly altered, which was not entirely consistent with reports in the literature (Vaiserman et al., 2017). However, the changes in the observed species, Chao1, Shannon, and Simpson indices still reflected the trend of recovery to the control group status with the increase in HDTO intervention doses. From the perspective of species level, as reported in literature, 80% of the microbiota detected in the aging model was divided into three dominant species: Firmicutes, Bacteroidetes, and Actinobacteria (Lay et al., 2005); the ratio of Firmicutes/Bacteroidetes in adults determined by q‐PCR was significantly lower than that of the elderly (Mariat et al., 2009). In our study, Bacteroidetes and Firmicutes were the dominant flora, accounting for more than 80% of the total population. However, their distribution was remarkably altered by injection of d‐galactose in mice. The abundance of Bacteroidetes decreased from 44.33% to 34.55%, while the abundance of Firmicutes increased from 39.73% to 54.52%; thus, the ratio of Firmicutes/Bacteroidetes increased significantly, while HDTO diet intervention restored all these abundance changes. The decrease in the abundance of Proteobacteria was inconsistent with the description in the literature on the change of this phylum abundance in the aging state, and when the HDTO supplement reached the medium dose, the abundance level of these bacteria were restored to that of the control. Shin et al. (2015) reported that Proteobacteria was a potential diagnostic marker of dysbiosis, and according to some current research, the response to dysbiosis was an increase in its abundance. However, it remains controversial whether in related dysbiosis, Proteobacteria abundance is increased or decreased, and its individual change can be used as a marker, which still needs to be further explored (Ma et al., 2020). Parabacteroides has also been reported to exclude potential pathogens from colonizing the gut, which is beneficial for the host (Yang et al., 2018). Deferribacteres have been proposed to be age related (Godoy‐Vitorino et al., 2010). HDTO supplementation at medium‐dose levels restored its abundance level to that of the control group. The abundance of Tenericutes increased significantly in the aging model, and this change was consistent with the results of the inflammatory aging model (Kim et al., 2016). Yang et al. (2020) postulated that the bacteria Tenericutes phylum might be one of the key players in gut microbiota alteration associated with cognitive function upon diet intervention. HDTO supplementation significantly prevented this increase.

An in‐depth study at the genus level can better understand which genera contribute to the alteration in the overall level of the phylum. The gut microbiota in the d‐galactose‐induced mice significantly increased the abundance of *Ligilactobacillus*, *Lactobacillus*, and *Erysipelothrix* of Firmicutes, and decreased the abundance of *Bacteroides* and *Alistipes* of Bacteroidetes, *Dielma* of Firmicutes and *Mucispirillum* of Deferribacteres. The metabolites that were markedly altered in the mimetic aging mice mainly included amino acids (l‐leucine, l‐methionine, l‐5‐oxoproline, l‐aspartic acid, glycine, proline, l‐valine, l‐threonine, tyrosine, l‐methionine, and l‐serine), sugars (d‐rhamnose, d‐erythritol, d‐xylose, 2‐α‐mannobiose, d‐arabinose, glucose, d‐galactose, l‐fucose, and d‐cellobiose), nucleic acids (uridine, pyrimidine, and uracil), organic acids (pyroglutamic acid, succinic acid, and lactic acid), alcohols (inositol and glycerol), and amphetamines. Amino acids are important building blocks required for protein synthesis and are essential for the synthesis of many bioactive molecules that participate in signal transduction, hormone production, reproduction, and muscle development (Dai et al., 2015). The levels of l‐serine and l‐threonine in the fecal metabolites of aging mice were significantly reduced, and glycine content increased. The inconsistent alterations in the levels of the three amino acids might reflect the disorder of the glycine–serine–threonine metabolic pathway, while glycine–serine–threonine metabolism is a pivotal metabolic hub related to life span (Aon et al., 2020). Branched chain amino acids (leucine, valine, isoleucine) play a key physiological role in regulating protein synthesis, metabolism, food intake, and aging (Le Couteur et al., 2020). l‐valine in the metabolites of d‐galactose‐induced aging mouse model was notably downregulated, and the level of l‐threonine was also significantly reduced. The biosynthetic pathway of valine–leucine–isoleucine may be disturbed. Uridine is a precursor of phospholipid synthesis, and its content is significantly reduced in the metabolites of model mice. Studies on elderly animals and neurodegenerative disease models have shown that combined supplementation of dietary phospholipid precursors (such as uridine, omega‐3 fatty acids, and choline) can increase cephalin, neurite outgrowth, synaptic proteins, dendritic spine formation, and nerve transfer (Perez‐Pardo et al., 2018). It has also been documented in the literature that phospholipids are associated with lower cholesterol levels (Ipsen et al., 1987; Ledreux et al., 2016). In our study, the levels of TC and LDL‐C were remarkably increased, the HDL‐C levels were notably decreased, and the mice showed abnormal serum lipid metabolism. This may be because d‐galactose disrupts uridine‐involved metabolism, thereby reducing the level of phospholipids and ultimately leading to an increase in cholesterol levels. Zhou et al. (2015) found that cholesterol levels in the liver of d‐galactose‐induced mice increased significantly. Compared with the model group, HDTO supplementation inordinately increased uridine levels; accordingly, serum cholesterol levels returned to the level of the control group. The microbial biomarkers *Bacteroides*, *Desulfovibrio*, and *Duncaniella* were negatively correlated with LDL‐C, Spiroplasma was significantly positively correlated with LDL‐C, *Bacteroides*, *Desulfovibrio*, and *Duncaniella* were positively correlated with HDL‐C, *Spiroplasma* was negatively correlated with HDL‐C, and *Erysipelothrix* was positively correlated with TC levels. 5‐Oxyproline is a product of glutathione metabolism, and is formed by the reduction of glutathione by glutamyl transpeptidase and glutamyl cyclotransferase. Glutathione is an antioxidant that removes reactive oxygen species produced by oxidative stress. The level of l‐5‐oxoproline in d‐galactose‐induced aging mice was significantly increased, owing to the possibility that d‐galactose induced a sudden increase in oxidative stress in mice and promoted the metabolism of glutathione. The content of l‐5‐oxoproline increased. However, when the supplemental dose of HDTO reached 260 mg/kg/day, its content in the fecal metabolites of d‐galactose‐induced aging mice returned to the level of the control group. The significant decrease in d‐arabinose in d‐galactose‐induced aging mice was related to a significant decrease in the abundance of Bacteroidetes in the intestine (Hor et al., 2019).

## 5 CONCLUSION

The modulatory effects of HDTO administration on aging mice were investigated. Behavioral experiments and serum biochemical indicators indicated that d‐galactose successfully induced senescence in mice, decreased memory and learning ability, increased blood lipid levels, and decreased antioxidant capacity. HDTO diet intervention significantly improved various aging manifestations. The abundance and diversity of gut microbiota in mice were elevated upon d‐galactose senescence induction. Specifically, at the phylum level, the abundance of Firmicutes and Tenericutes was significantly increased, while that of Bacteroidetes, Proteobacteria, and Deferribacteres was significantly decreased. At the genus level, the variation mainly presented as an increase in the abundance of the Firmicutes genera *Ligilactobacillus*, *Lactobacillus*, and *Erysipelothrix*, the decrease in the abundance of Bacteroidetes genera *Bacteroides* and *Alistipes*, Firmicutes genus *Dielma*, and Deferribacteres genus *Mucispirillum*. The significantly differential gut microbiota showed an obvious correlation with its metabolites and aging‐related indices. Meanwhile, d‐galactose resulted in disorders of eight metabolic pathways, and the metabolites with obvious changes mainly included amino acids, sugars, and uridine. HDTO diet intervention partially reversed the above alterations, and the reversal effect was different with the HDTO dose.

## Supporting information


Figure S1

Figure S2

Table S1
