# Supplementary figures and images for: Gut microbiome and metabolome analyses reveal the protective effect of special high‐docosahexaenoic acid tuna oil on d‐galactose‐induced aging in mice
Source: Food Sci Nutr. 2022 Jul 15;10(11):3814–27. doi: 10.1002/fsn3.2978 (PMC9632196; doi:10.1002/fsn3.2978)

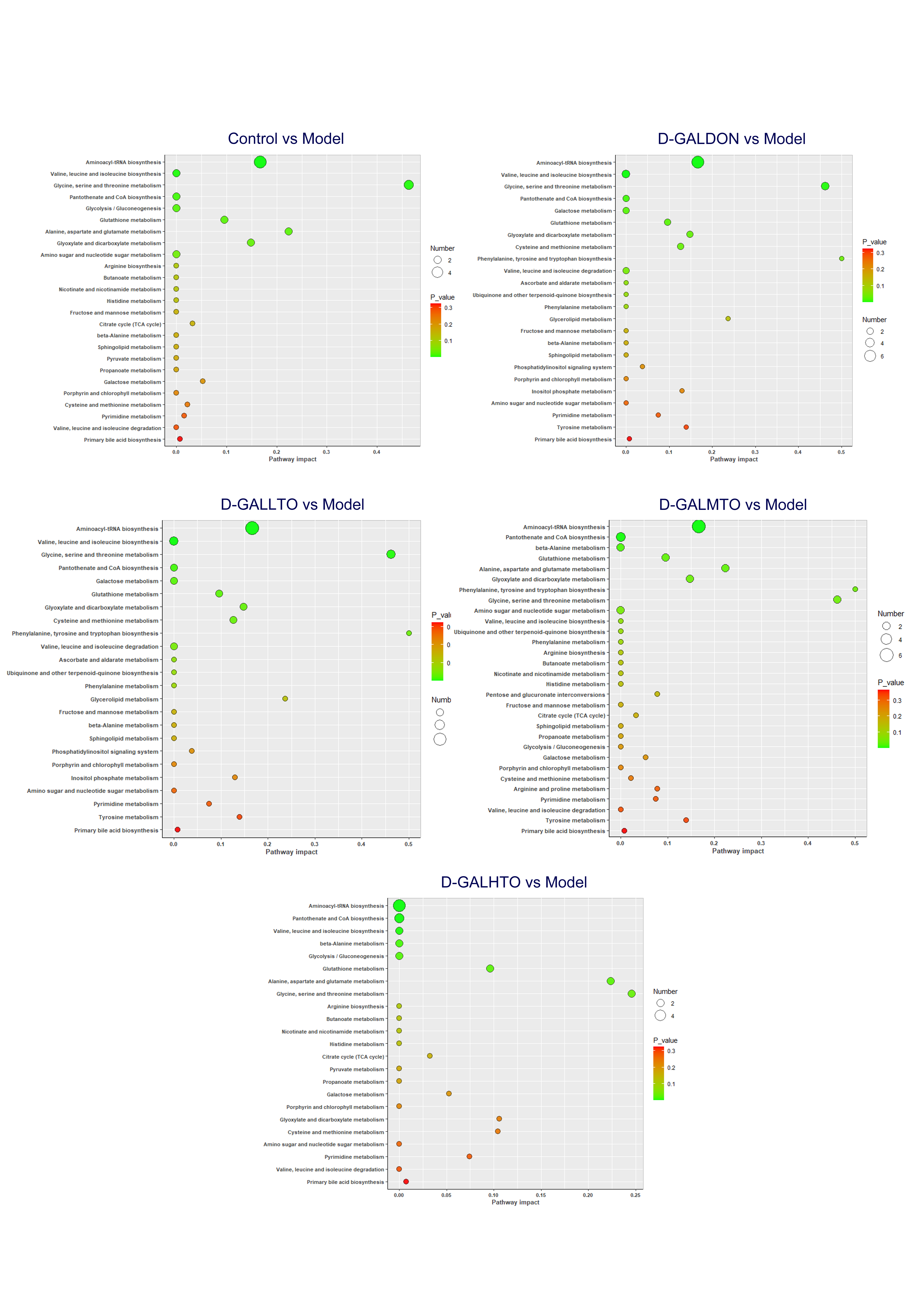

Supplement: Supplementary file 2 — Figure S2 [file FSN3-10-3814-s001.tif]
