# Supplementary material for: Gut microbiome and metabolome analyses reveal the protective effect of special high‐docosahexaenoic acid tuna oil on d‐galactose‐induced aging in mice
Source: Food Sci Nutr. 2022 Jul 15;10(11):3814–27. doi: 10.1002/fsn3.2978 (PMC9632196; doi:10.1002/fsn3.2978)
Supplement: Supplementary file 3 — Table S1 [file FSN3-10-3814-s003.docx]

**Table S1** Fatty acid profile in the HDTO

| **Fatty acid** | **HDTO (%)** | **TO (%)** |  | **Fatty acid** | **HDTO (%)** | **TO (%)** |
| --- | --- | --- | --- | --- | --- | --- |
| C_14:0_ | 3.61±0.44 | 3.90±0.23 |  | C_20:2_ | ND | 1.40±0.17 |
| C_15:0_ | 1.20±0.08 | 1.39±0.41 |  | C_18:2_ | 0.69±0.06 | ND |
| C_16:0_ | 16.85±0.29 | 26.74±0.01 |  | C_17:1_ | ND | 0.74±0.09 |
| C_16:1_ | 5.42±0.16 | 5.61±0.34 |  | C_19:0_ | 1.37±0.23 | 0.80±0.10 |
| C_18:0_ | ND | 0.40±0.21 |  | C_20:5_ | 1.29±0.00 | 0.80±0.06 |
| C_19:2_ | ND | 0.23±0.55 |  | C_20:4_ | 3.48±0.13 | 2.14±0.05 |
| C_17:1_ | 2.60±0.24 | 1.68±0.33 |  | C_20:5_ | 7.42±0.11 | 5.21±0.17 |
| C_17:0_ | 1.62±0.34 | 1.62±0.06 |  | C_16:0_ | 1.15±0.23 | ND |
| C_17:1_ | 0.58±0.03 | 0.80±0.03 |  | C_22:5_ | 4.17±0.20 | 2.00±0.13 |
| C_19:0_ | 5.72±0.73 | 6.50±0.33 |  | C_21:5_ | 1.23±0.15 | 0.65±0.04 |
| C_18:1_ | ND | 17.45±0.61 |  | C_22:6_ | 40.30±0.23 | 20.07±0.41 |
| C_24:1_ | 1.22±0.08 | ND |  |  |  |  |

* ND, not detected in this tuna oil.
